# Pathways Relating the Neurobiology of Attachment to Drug Addiction

**DOI:** 10.3389/fpsyt.2019.00737

**Published:** 2019-11-08

**Authors:** Lane Strathearn, Carol E. Mertens, Linda Mayes, Helena Rutherford, Purva Rajhans, Guifeng Xu, Marc N. Potenza, Sohye Kim

**Affiliations:** ^1^Attachment and Neurodevelopment Laboratory, Stead Family Department of Pediatrics, University of Iowa Carver College of Medicine, Iowa City, IA, United States; ^2^Center for Disabilities and Development, University of Iowa Stead Family Children’s Hospital, Iowa City, IA, United States; ^3^Yale Child Study Center, Yale University School of Medicine, Yale University, New Haven, CT, United States; ^4^Department of Pediatrics, Baylor College of Medicine, Houston, TX, United States; ^5^Department of Epidemiology, College of Public Health, University of Iowa, Iowa City, IA, United States; ^6^Departments of Psychiatry and Neuroscience and the National Connecticut Mental Health Center, Yale University, New Haven, CT, United States; ^7^Department of Obstetrics and Gynecology, Baylor College of Medicine, Houston, TX, United States; ^8^Menninger Department of Psychiatry and Behavioral Sciences, Baylor College of Medicine, Houston, TX, United States

**Keywords:** attachment, addiction, oxytocin, dopamine, glucocorticoid, adverse childhood experiences

## Abstract

Substance use disorders constitute a significant public health problem in North America and worldwide. Specifically, substance addictions in women during pregnancy or in the postpartum period have adverse effects not only on the mother, but also on mother-infant attachment and the child’s subsequent development. Additionally, there is growing evidence suggesting that parental addiction may be transmitted intergenerationally, where the child of parents with addiction problems is more likely to experience addiction as an adult. The current review takes a developmental perspective and draws from animal and human studies to examine how compromised early experience, including insecure attachment, early abuse/neglect, and unresolved trauma, may influence the development of neurobiological pathways associated with addictions, ultimately increasing one’s susceptibility to addictions later in life. We approach this from three different levels: molecular, neuroendocrine and behavioral; and examine the oxytocin affiliation system, dopamine reward system, and glucocorticoid stress response system in this regard. Increased understanding of these underlying mechanisms may help identify key targets for early prevention efforts and inform needed intervention strategies related to both insecure attachment and addiction.

## Introduction

Within the United States and throughout the world, substance addiction is a significant problem with wide-ranging implications. Substance-use disorders in North America are a growing public health crisis with associated costs reported as reaching billions of dollars annually (1). According to the National Institute on Drug Abuse, the abuse of tobacco, alcohol, and illicit drugs in the United States tops more than $740 billion annually in costs related to crime, loss of work productivity, and health care (2). Aside from these monetary costs, the disruption to the development of secure attachment in children living in environments with substance abusing parent(s) may result in substantial risk to children, parents, and society. Of further concern is the paucity of effective treatment options, which are usually focused on the current addiction behavior rather than the underlying experiences and mechanisms that may have predisposed the individual to addiction (3).

Substance-abusing women who are either pregnant and/or have children face a significant challenge, with their addiction associated with many potential long-term adverse consequences impacting their children. Nearly 90% of women who struggle with substance-use disorders are of reproductive age (4). Although some women may abstain from substances during pregnancy, many resume substance use during the postpartum period, with adverse effects on their parenting capacities and their children’s developmental trajectories. Indeed, some have suggested that the stress associated with parenting may become a risk factor for relapse in substance-using parents (5).

Addictions in mothers are associated with a range of parenting difficulties and may sometimes involve child abuse and neglect (6, 7). Ultimately, the ramifications may include having their children placed in foster care, which may further compromise the quality of the parent-child attachment. Mothers with addictions are considerably more likely than those without addictions to lose custody of their children as a result of child neglect or abuse (8). Further, an expanding body of research supports the notion that parental addiction may be transmitted intergenerationally through several possible mechanisms, with the child more likely to experience addiction as an adult (3, 9).

Twenty years ago, based on the accumulation of brain imaging research, it was argued that addiction should be defined as a brain disease, rather than solely a social or societal problem (10). While still acknowledging crucial behavioral and social-context components, this model characterized addiction as the result of repeated exposure to drugs of abuse, causing changes in brain structure and function related to reward experience and anticipation, perception and memory, and cognitive control. This model accentuated the importance of treatments that incorporated biological, as well as behavioral and societal approaches.

More recently, however, this widely accepted model has been challenged (11–13), with some authors advocating a “developmental-learning model” which characterizes addiction as a product of cognitive and emotional development, particularly during early childhood and adolescence. Lewis (12, 13) provides a specific neurobiological account of how experience and learning—particularly in an environment of chronic stress—may alter neural development and connectivity leading to addiction *via* the normal mechanisms of neuroplasticity, producing a ventral-to-dorsal shift in striatal activation with more compulsive behavior, and diminished cortical control *via* the dorsolateral prefrontal cortex.

**Figure 1:**
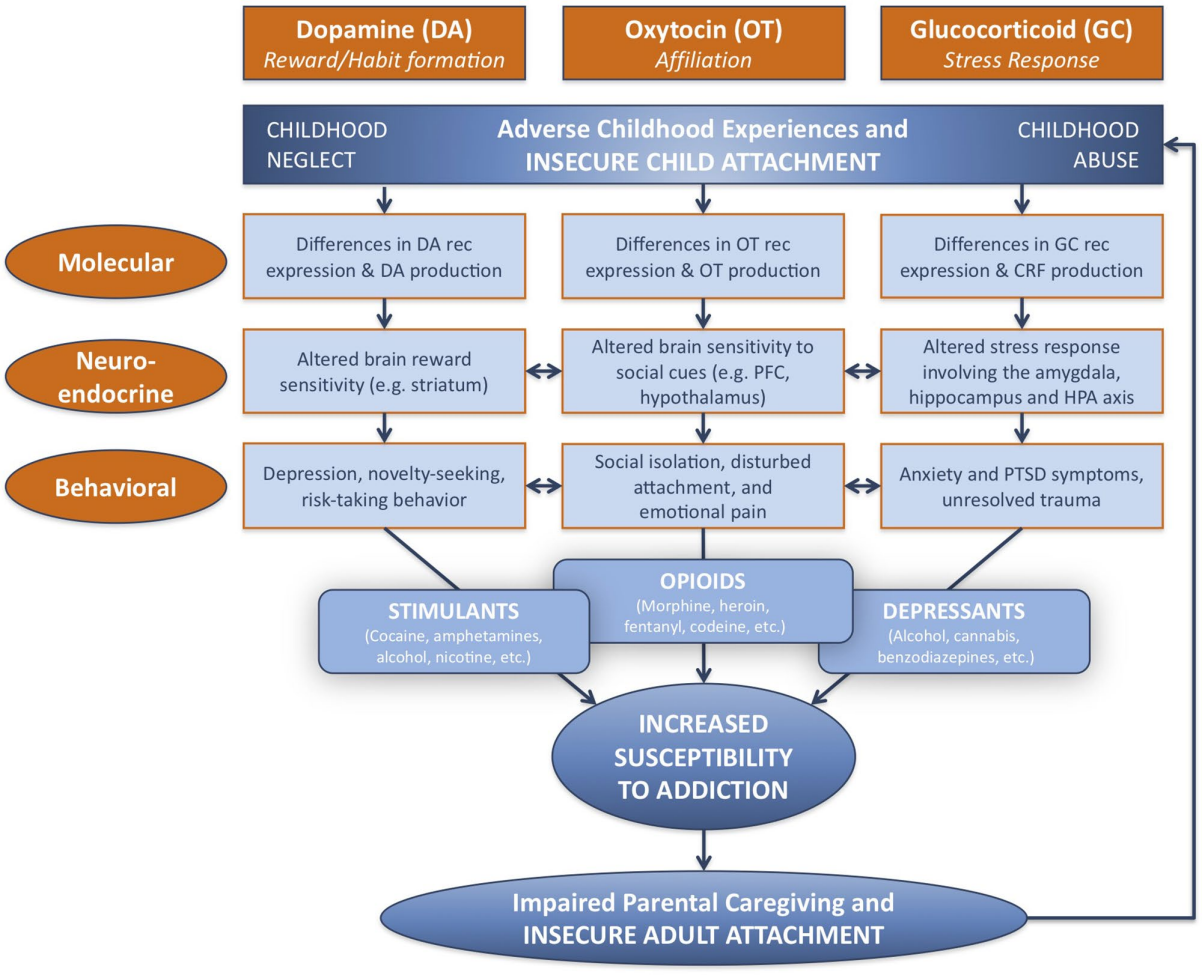
Developmental and neurobiological pathways linking adverse childhood experience to susceptibility to addiction, *via* modifications in dopamine-related, oxytocin-related, and glucocorticoid-related systems at molecular, neuroendocrine, and behavioral levels. Childhood adversity, including abuse and neglect, may be associated with insecure attachment, and lead to behavioral patterns linked with specific patterns of substance abuse. Parental addiction may impair parental caregiving capacity as a result of insecure patterns of adult attachment, and perpetuate the cycle of childhood adversity and addiction. DA, dopamine; OT, oxytocin; GC, glucocorticoid; rec, receptor; CRF, corticotropin-releasing factor; PFC, prefrontal cortex; HPA, hypothalamic–pituitary–adrenal; PTSD, post-traumatic stress disorder. Adapted from (3) © 2016 New York Academy of Sciences. Used with permission from John Wiley and Sons.

Investigating and understanding the relationship between attachment and substance addiction is of utmost importance. Here, the neural mechanisms underlying this relationship will be considered at three different levels: molecular, neuroendocrine, and behavioral, in terms of major biological systems associated with each. These include the dopamine-related reward/reinforcement/habit formation system, the oxytocin-related affiliation system, and the glucocorticoid-related stress response system (**Figure 1**).

## Types of Addiction

For the purposes of this manuscript, addiction will be restricted to substance-use disorders as defined by criteria in the fifth edition of the Diagnostic and Statistical Manual (DSM-5). It is important to note that the DSM-5 and the recently approved eleventh edition of the International Classification of Diseases (ICD-11) include non-substance or behavioral addictions (e.g., gambling disorder), and existing data indicate negative impacts of gambling disorder on children (14). Gambling disorder has also been associated with insecure attachment styles, suggesting that early life experiences relating to attachment are important to consider in behavioral addictions (15). Substance-use disorders, especially those at the more severe end of the spectrum, are psychiatric conditions characterized by habitual and pathological patterns of drug-seeking and drug-consuming behaviors (16). Habitual patterns of drug-seeking and drug consumption absorb a large amount of time and attention in drug addictions, leading to significant functional impairment in meeting responsibilities at home, school and work (16). When abstaining from the use of a drug or chemical substance, symptoms of distress and strong urges or cravings to use the substance again may emerge and become particularly salient (9). In addition to substance addiction, behavioral addictions to activities such as gambling, gaming and potentially other behaviors (sex, specific types and patterns of internet use) share parallel features with substance addictions: 1) continuing engagement in the addictive behavior despite adverse outcomes; 2) compulsive engagement in the addictive behavior; 3) a craving or appetitive urge state prior to the engagement in the addictive behavior; and 4) diminished control over engagement in the addictive behavior (17, 18). While less research has investigated the impact of behavioral addictions (as compared to substance addictions) on parenting and attachment, features of substance addictions parallel behavioral addictions, suggesting that behavioral addictions may also interfere with parent–child attachment.

While substance use may influence the brain and behavior, it is still unclear why some individuals struggle with addiction and others do not. Once a person has used a particular substance, it may or may not lead to addiction. The developmental-learning model of addiction proposes that early experience may alter susceptibilities to different types of addiction through changes in specific neural circuits. Along this line, Alvarez-Monjaras and colleagues (2018) (9) have integrated neurobiological and psychodynamic theories through the lens of attachment: the neurobiological approach centers on identifying biological mechanisms that may influence the development of substance use and addiction while the psychodynamic approach provides a framework for understanding relational and representational aspects of addiction within a developmental perspective.

Attachment has been defined by Ainsworth (19) as a tie that endures across time and space, to a particular person to whom one turns when feeling vulnerable or in need of protection from danger. John Bowlby (20–22) introduced attachment theory as a structural, systemic model focused on the function and development of human protective behavior. Bowlby’s attachment theory asserts that humans are inherently predisposed to form attachment relationships to their primary caregivers, particularly the mother. These attachment relationships serve to protect the child and occur in an organized form by the end of the first year of life (23). According to the Dynamic-Maturational Model of Attachment and Adaptation (23), the organization of attachment continues across one’s lifetime as a means of: 1) adapting to adverse environments and strategically protecting oneself from danger; and 2) ensuring reproductive success. Individuals who are exposed to danger, either from direct threat or absence of care, particularly during infancy, are more likely to distort or negate cognitive and affective information, and thus misinterpret crucial information from their environment. This may help to explain seemingly irrational decisions made by individuals with substance addictions as they attempt to secure a sensation of reward, gain social affiliation, or reduce stress by using substances.

More recently, with advances in neuroimaging, attachment has been systematically associated with neuroendocrine responses to salient attachment cues, such as a mother’s response to seeing her baby’s smiling or crying face. Such neural and endocrine responses involve activation of the dopamine-related reward system, oxytocin-related affiliation system, and glucocorticoid-related stress-response system (24), which pathways are also implicated in the neurobiology of drug addiction.

## Patterns of Attachment

Since Bowlby first published his seminal work (20), numerous methods have been developed to systematically classify patterns of attachment, as observed from infancy to adulthood. Ainsworth (19) initially identified and empirically linked three major patterns of attachment with their origins in maternal responsiveness and sensitivity: Secure (Type B), Avoidant (Type A), and Ambivalent (Type C). This has led to the development of what has become one of the most accepted and empirically tested measures of attachment in adulthood: the Adult Attachment Interview (AAI) (25). The AAI is a semi-structured interview designed to identify differences in *state of mind with regard to overall attachment history* by examining participants’ abilities to describe attachment-related memories while simultaneously maintaining coherent, cooperative discourses (26). The results of a recent longitudinal meta-analysis of 34 samples (total N of 56,721) confirmed a significant association between insecure attachment and substance-abuse problems (27).

The Dynamic-Maturational Model of Attachment and Adaptation (23) involves a modification of the original AAI, extending Ainsworth’s childhood classifications into adulthood. This model, which has a broader focus on psychopathology and trauma, may be particularly suited for understanding high-risk populations including those of substance-using individuals (28). From an analysis of the transcribed discourse, four basic attachment patterns emerge among adults, summarized as secure (Type B1-3), “insecure/dismissing” (Type A1-6), “insecure/preoccupied” (Type C1-6), and “insecure/mixed” (Type A/C). Longitudinal studies have suggested the unique capacity of a caregiver’s AAI to predict attachment patterns in the infant offspring (29, 30). Understanding the neurobiological differences between attachment patterns among adults may help us better understand the mechanisms underlying the intergenerational transmission of addictions.

Crittenden has suggested that basic attachment patterns may, in fact, represent differences in how the brain processes sensory information (31). Accordingly, she proposed that sensory stimulation is transformed into one of two basic forms of information: 1) temporally ordered “cognitive” information and 2) intensity-based arousal or “affective” information. The first is proposed to be the predominant mechanism in “dismissing” attachment organization, whereas the second is proposed to be central in “preoccupied” attachment. “Secure” organization may involve a balanced integration of both sources of information. For example, “dismissing” adults tend to dismiss their own feelings, intentions and perspectives and rely more upon rules and learned temporal relations in predicting future rewards. “Preoccupied” adults, in contrast, may organize their behavior around affective information, such as fear, anger or desire for comfort. They tend to be preoccupied by their own feelings and perspectives, while omitting or distorting cognitive or temporally ordered information. Adults with “secure” or balanced patterns of attachment may be best able to integrate temporally ordered information regarding causal effects, as well as more affect-based information, such as emotional states and imaged memory, in order to form close relationships, make accurate decisions and predict future reward. The organization of attachment may also involve the differential development of specific memory systems within the brain, such as procedural and semantic memory, imaged memory, and episodic and working memory systems, each of which is specifically coded in the AAI as considered in the Dynamic-Maturational Model, and linked to the function of specific brain regions and networks (23).

**Figure 2:**
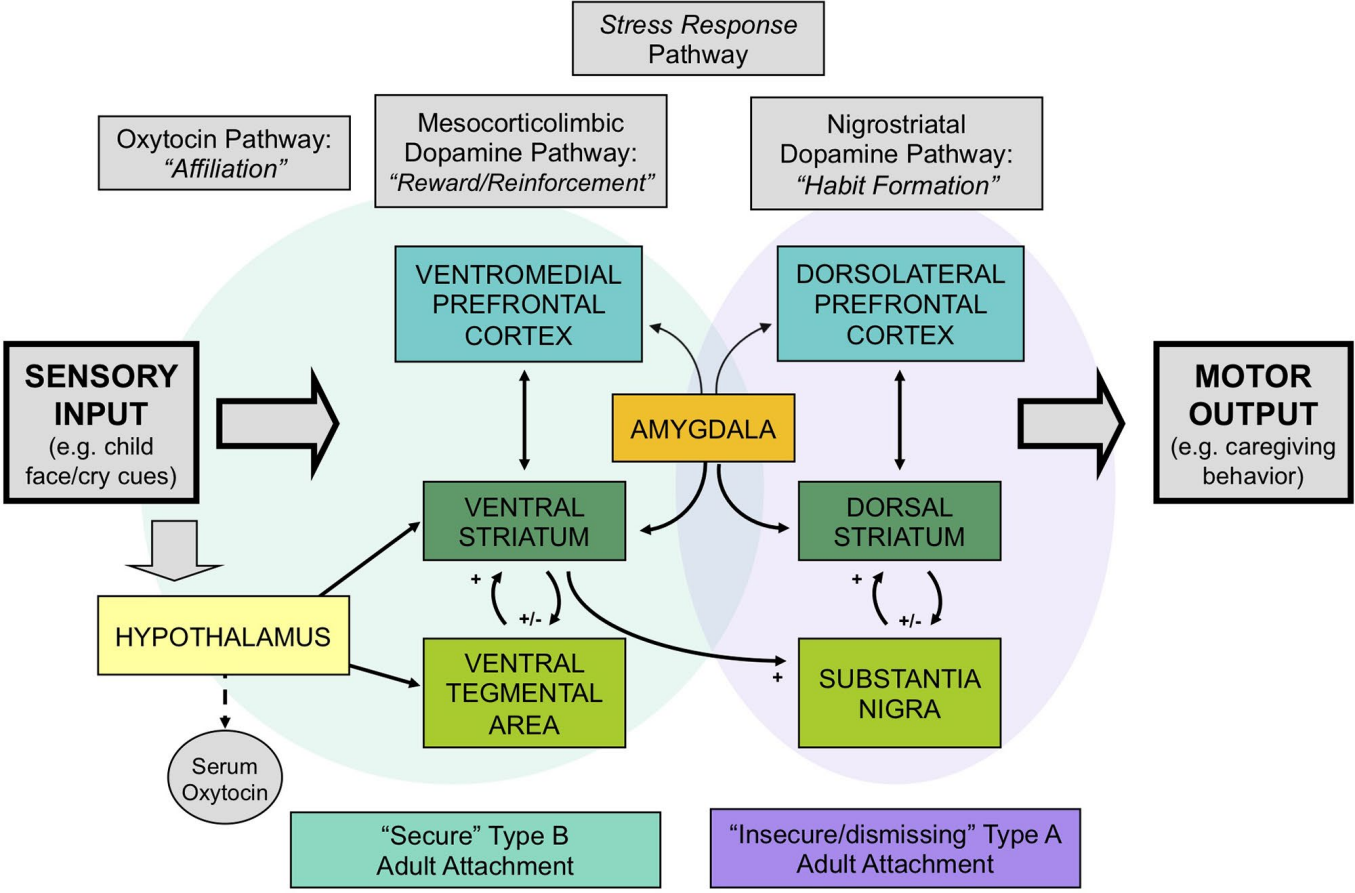
The neurobiology of attachment, incorporating 1) dopamine-related “reward/reinforcement” and “habit” pathways, 2) oxytocin-related “affiliation” pathways, and 3) glucocorticoid-related stress-response pathways. Secure patterns of attachment are associated with greater activation of the mesocorticolimbic and oxytocin-associated circuits, whereas insecure/dismissing attachment is associated with greater activation of nigrostriatal dopamine pathways. Adapted from (36) © 2011 The Author, *Journal of Neuroendocrinology*. © 2011 Blackwell Publishing Ltd. Used with permission from John Wiley and Sons.

Two neuroendocrine systems that appear to be related to Crittenden’s theory of cognitive and affective forms of information processing and attachment are the dopamine and oxytocin systems. These neurodevelopmental processes appear to be shaped by early-life experience, including variations in maternal behavior (32–35) (**Figure 1**). The dopamine system includes two main components: the mesocorticolimbic and nigrostriatal dopamine pathways (**Figure 2**). The former involves stimulus-reward learning and prospective decision-making based upon predicted reward (37); the latter is implicated in motoric behaviors and habit formation. The oxytocin system is important in prosocial affiliative behavior, the formation of social and spatial memories, and emotion regulation (38). Oxytocin neurons connect the hypothalamus with the mesocorticolimbic dopamine system, including the ventral tegmental area and the ventral striatum, and may facilitate reward responses and reinforcement to affective and social cues. Recent data have suggested interactive relationships between the mesocorticolimbic dopamine, oxytocin and glucocorticoid physiological stress systems (34, 39). Oxytocin receptor blockade gives rise to both an exaggerated adrenocorticotropic hormone (ACTH) as well as corticosterone stress hormone response in rats (40). Similar results are observed in oxytocin-deficient knock-out mice (41). Working alongside the oxytocin system, the mesocorticolimbic dopamine system similarly has a stress inhibitory effect on the amygdala (42) through the medial prefrontal cortex (43). Consequently, it appears that one important function of these inter-related neuroendocrine systems may be to modulate human stress responses and thereby facilitate optimal social bonding and attachment, through different but complementary mechanisms.

Our research suggests that differences in human attachment strategies are associated with significant differences in these brain activation patterns, when mothers view powerful visual stimuli that may impact the attachment system: images of their own infant’s face (24) (**Figure 2**). Mothers with secure (Type B) patterns of attachment, compared with insecure/dismissing (Type A) mothers, showed greater activation of regions in the mesocorticolimbic dopamine pathway, including the ventral striatum and ventromedial prefrontal cortex, and the oxytocin-associated hypothalamic/pituitary region. Mothers with Type A attachment, in general, showed greater activation of the dorsolateral prefrontal cortex, which has been implicated in cognitive control and habit formation.

## Fathers and Attachment

Most early investigations of parent–child attachment focused solely on the mother-child attachment relationship, overlooking the father as an essential element in the child’s attachment formation process. According to Scism and Cobb (2017), the importance of creating an immediate mother-infant bond overshadowed and deferred the efforts of researchers to document factors and interventions that influence father–infant bonding. In the late 1970s and early 1980s, investigators began to recognize the importance of paternal involvement during the immediate postpartum period (44, 45), leading to additional weight gain in preterm infants, reducing cognitive delays in offspring, and increasing breastfeeding rates in mothers (46, 47).

In recent years, the influence of father–infant interactions on attachment has become a focus of research within the field of child development, showing, for example, that fathers have a unique but complementary neuroendocrine response to infant interactions, compared with mothers (48). Much evidence has indicated that fathers are critical for the well-being of their children (49, 50) and that the absence of fathers is associated with numerous risk conditions (51). Observational investigations of the nature of parental involvement have found that father–child interaction patterns have a distinctive quality that is more dynamic and stimulating than mother-child interaction patterns, which may help promote exploration and risk-taking behaviors and ultimately facilitate cognitive development (49). Although some preliminary studies have explored the effect of parenting interventions in fathers with addiction problems (52), little is known about the direct role of fathers in preventing later addiction problems in their children (53).

## Pathways to Addiction

Proposed pathways leading to addiction are numerous and multifaceted, involving expression of specific molecular and genetic entities, altered brain sensitivities to reward- and stress-related cues, environmental influences, and cognitive, behavioral, motivational and emotional constructs that include depression, risk-taking, social isolation, emotional pain and/or unresolved trauma (3) (**Figure 1**). Epidemiological studies have shown associations between adverse early childhood experience and addictions in adolescence and adulthood. For example, after adjusting for multiple sociodemographic and potential confounding variables, individuals who had experienced childhood abuse and/or neglect were more likely to use tobacco and alcohol in early adolescence, become dependent on cannabis, and smoke and inject drugs in early adulthood (54–57). Parental figures who abuse drugs have often experienced inadequate caregiving environments during their own childhoods (9). Furthermore, their own substance addiction increases the likelihood that they will provide neglectful or abusive care to their own children (58). This may also lead to children being placed in foster care, further impacting parent-child attachment.

Negative childhood experiences often have a profound and enduring influence upon the developing child’s quality of life and well-being, frequently well into adulthood. The Adverse Childhood Experiences (ACEs) Study has examined retrospectively recalled traumatic experiences that occurred during the first 18 years of life (59, 60). ACE categories include multiple forms of abuse (physical, emotional, and sexual), neglect (physical and emotional), parental separation or divorce, household violence, substance use, mental illness, and incarceration (59). An examination of the relationship between illicit drug use and ACEs found that each ACE experience increased the likelihood of early initiation of drug use 2- to 4-fold, while people with ≥5 ACEs were 7- to 10-fold more likely to report illicit drug use, addiction to illicit drugs, and drug use by parents (60, 61).

A substantial threat to healthy development is growing up in poverty. According to the KIDS COUNT Data Book (2018), a national average of 19% (14.1 million) of children in the United States lived in poverty in 2016, with some states reaching as high as 30%. Notably, poverty rates vary tremendously according to race, with African-American and American Indian children (both 34%) experiencing nearly three times the poverty rate for Caucasian and Asian and Pacific Islander (both 12%). Employment insecurity and high rates of job instability may disrupt daily living and relationships and compromise families’ abilities to invest in their children’s development. This may lead to diminished achievement in school and reduced likelihoods of future success. Secure employment is a significant pathway to financial equilibrium and well-being in families. However, Koball and Jiang (62) note that being a child in a low-income family does not happen by chance. Factors related to poverty include parental education, parental employment, race and ethnicity, family structure, region of residence, residential instability, and utility and housing insecurity. Children and parents who live in high-poverty neighborhoods face many challenges that may impact their lives on a daily basis including greater financial instability, poorer health, higher rates of violence and crime, poorer schools, and limited access to support systems and job opportunities. Each of these challenges may add to the stress level of both the child and the parent, which may interfere with attachment relationships and predispose the offspring to addiction (63).

Understanding the mechanisms by which early adverse experience may increase susceptibility to addiction is of critical importance to adequately formulating plans for prevention and treatment. Three neurobiological pathways have been identified that may link attachment and early experience with addiction at molecular, neuroendocrine and behavioral levels. These pathways include: 1) the dopamine-related reward system; 2) the oxytocin-related affiliation system; and, 3) the glucocorticoid-related stress response system (3). Each of these systems will be outlined below, summarizing what we understand about the neurobiology of attachment and how this may relate to addiction behaviors and susceptibility.

### Oxytocin-Related Affiliation System Pathways

Oxytocin is a neuropeptide that functions as a neuro-regulator of social behavior within mammalian species (64). Considerable attention has been focused on oxytocin’s core roles in attachment formation and stress regulation, and there has been a surge of interest in the connection between dysregulated oxytocin systems and disorders of psychosocial functions (65–67). In particular, the developing oxytocin system has been implicated in increasing vulnerability to addiction across the lifespan (68) as well as serving a protective function against the development of addiction (69). Specifically, oxytocin may enhance the salience and familiarity of social cues and lessen novelty- and reward-seeking, which have been implicated in pathways to addiction (69). Drug use may also impact the maternal oxytocin system. In postpartum human mothers, cocaine exposure during pregnancy was associated with decreased oxytocin in plasma relative to mothers not using substances during pregnancy (70). In rodent dams, chronic cocaine exposure during pregnancy also resulted in decreased oxytocin levels in the maternal brain, including in the hippocampus, ventral tegmental area, and medial preoptic area (71). Therefore, the oxytocin system may be implicated in addiction susceptibility before the transition to motherhood, and may be modulated by drug exposure during pregnancy and the postpartum period.

The oxytocin system may be of particular significance in relation to addiction and attachment because of its neuroplasticity in response to an individual’s early social environment (69). Growing evidence suggests early life experiences may substantially impact long-term functioning of the oxytocin system (24, 72, 73). On the behavioral level, rat pups experiencing early-life deprivation show multiple social impairments including diminished social motivation (74), reduced affiliative behavior (75), impaired social learning (76) and increased aggressive behavior (74, 77). Rat pups who received low levels of care early in life tended to develop into adults that display similarly low levels of care with their own pups (78, 79). This intergenerational transmission appears to be at least partially mediated by oxytocin-related molecular and neuroendocrine alterations, including changes in oxytocin receptor expression and oxytocin production (80, 81) (**Figure 1**).

Empirical evidence from human research parallels findings from animal models. Extreme early deprivation in humans has been prospectively associated with severe long-term attachment disorders and social deficits (81). Numerous longitudinal and cross-sectional studies have linked early trauma and/or disrupted attachment to long-term social and attachment difficulties (82–85). Other studies have linked compromised oxytocin functioning with social deficits (65, 86, 87). In humans, social isolation and low social support may accelerate the emergence and recurrence of substance use and predict substance addiction (88).

A history of early childhood trauma or stress has been negatively correlated with levels of oxytocin as assessed in cerebrospinal fluid, urine, or plasma (89–92). A dose-dependent inverse relationship has been observed between the severity of trauma experiences and oxytocin concentration (65, 68). Among the different types of trauma, emotional abuse and neglect appear to have the strongest associations (90, 91). Since adverse experiences in childhood (rather than adolescence and adulthood) emerge as arguably the most robust predictors of long-term oxytocin functioning, the timing of the trauma and adversity appears critical to the impact that oxytocin may have upon individual functioning (92).

**Figure 3:**
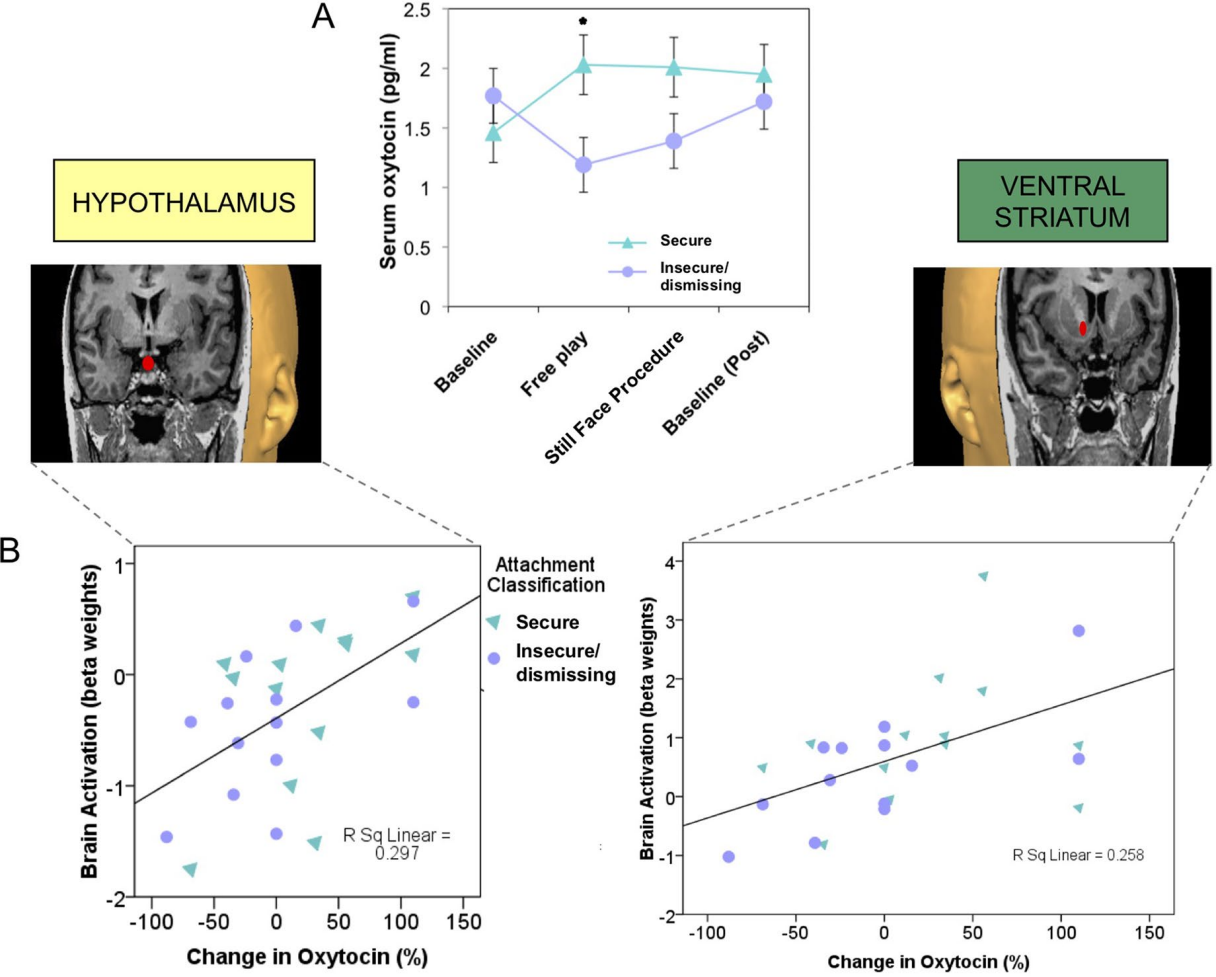
Plasma oxytocin response to mother–infant interaction is reduced in mothers with insecure/dismissing attachment, compared with securely attached mothers **(A)**, and is correlated with activation of hypothalamus (*r*_s_ = 0.6, *p* = 0.001) and ventral striatum (*r*_s_ = 0.57, *p* = 0.001), in response to viewing own-infant faces in a functional MRI scanner **(B)**. (36) Adapted from (24) © 2011 The Author, *Journal of Neuroendocrinology*. © 2011 Blackwell Publishing Ltd. Used with permission from John Wiley and Sons.

Infants or children who experience less synchronous, less sensitive, or less responsive caregiving show blunted salivary oxytocin levels, both at baseline and in response to social cues (72, 93), as well as disrupted patterns of attachment (94, 95). Adults with an insecure/dismissing (Type A) pattern of attachment often have a diminished peripheral oxytocin response when interacting with their infants (**Figure 3A**), which is correlated with reduced brain activation in oxytocin- and dopamine-associated brain regions, including the hypothalamus and ventral striatum (24) (**Figure 3B**). Peripheral oxytocin response (between a baseline of mother-infant separation and an interaction period) was also associated with differences in maternal behavior. A lower (or negative) oxytocin response was associated with diminished maternal gaze toward her infant, especially during heightened infant distress (86).

Studies are currently underway to test whether oxytocin may be an adjunctive treatment alongside methadone maintenance therapy for opioid-use disorders (96, 97). According to the brain opioid theory of social attachment (98), endogenous opioids are released in response to social bonding experiences, including social touch and breastfeeding. Social isolation may lead to reduced opioid activity and subsequent feelings of distress and emotional pain relating to separation and loss. Rising rates of opioid abuse and overdose deaths may be one consequence of disrupted “social capital” in society (99), mediated *via* changes in the oxytocin affiliation system.

### Dopamine-Related Reward System Pathways

The dopamine-related reward system contributes to the regulation of reward, motivation, and decision-making. The majority of dopamine neurons are located in the ventral part of the midbrain (100), where the mesocorticolimbic and nigrostriatal dopamine systems originate (**Figure 2**). Dopamine-related dysfunction has been associated with the pathophysiology of many psychiatric disorders, including depression and substance addictions. While multiple studies have reported abnormal dopamine-related functioning in addiction (101, 102), including decreased striatal dopamine receptor availability and dopamine release, these differences are seen primarily in stimulant and alcohol abuse, rather than abuse of opioids and cannabis (103) (**Figure 1**).

A significant and growing body of research has established the role of early-life experience in shaping the development of dopamine-related systems. Animal models have shown that early adverse experience alters dopamine-related neuronal activity and synaptic functions. For example, rat pups that are reared in isolation with prolonged maternal separation, show reduced dopamine transporter binding in the ventral striatum, increased baseline dopamine levels, and exaggerated dopamine release in response to acute stress (34, 104). Rodent studies investigating naturally occurring variations in maternal behavior (i.e., licking/grooming in rats) have demonstrated that diminished dopamine release in the ventral striatum leads to decreased licking and grooming of the rat pups in response to pup vocalization (32). High levels of postnatal maternal care provided to rat pups have been associated with an increased density of dopaminergic cell bodies within the ventral tegmental area and increased dopamine receptor mRNA levels within the ventral striatum (**Figure 2**). This association appears to persist into adulthood (105).

Notably, early adverse experience may also impact levels of stimulus-evoked dopamine release. Rodents with early adversity show dampened dopamine release in the ventral striatum in response to pups (106), enhanced dopamine release in the ventral striatum and hypothalamus in response to stress (107), and enhanced dopamine release in the ventral striatum in response to the administration of amphetamine (108). More specifically, suboptimal early caregiving in humans has been correlated with elevated dopamine release in the ventral striatum in response to stress (35) and amphetamine administration (109–111).

Behaviorally, rat-pups subjected to early maternal deprivation are more sensitive to novel stimuli in adulthood, an indicator of their enhanced spontaneous locomotor activity in novel settings (111, 112). In humans, novelty-seeking, or sensation-seeking, is defined by an amplified tendency toward novel sensations and experiences, often leading to impulsive risk-taking and/or the active pursuit of rewards (113). The link between early adversity and novelty-seeking has been observed in both community (114) and high-risk samples (115). Among different classes of substances, individuals with high novelty-seeking behavior display a preference for stimulant drugs that activate dopamine-related pathways (116). In a study of a large sample presenting with a complex set of risk factors, novelty-seeking emerged as the strongest factor contributing to the development of substance-related disorders (115). The link between childhood adversity and the presence of substance-use disorders was found to be at least partially mediated by increased novelty-seeking in individuals with histories of adverse life events (115).

**Figure 4:**
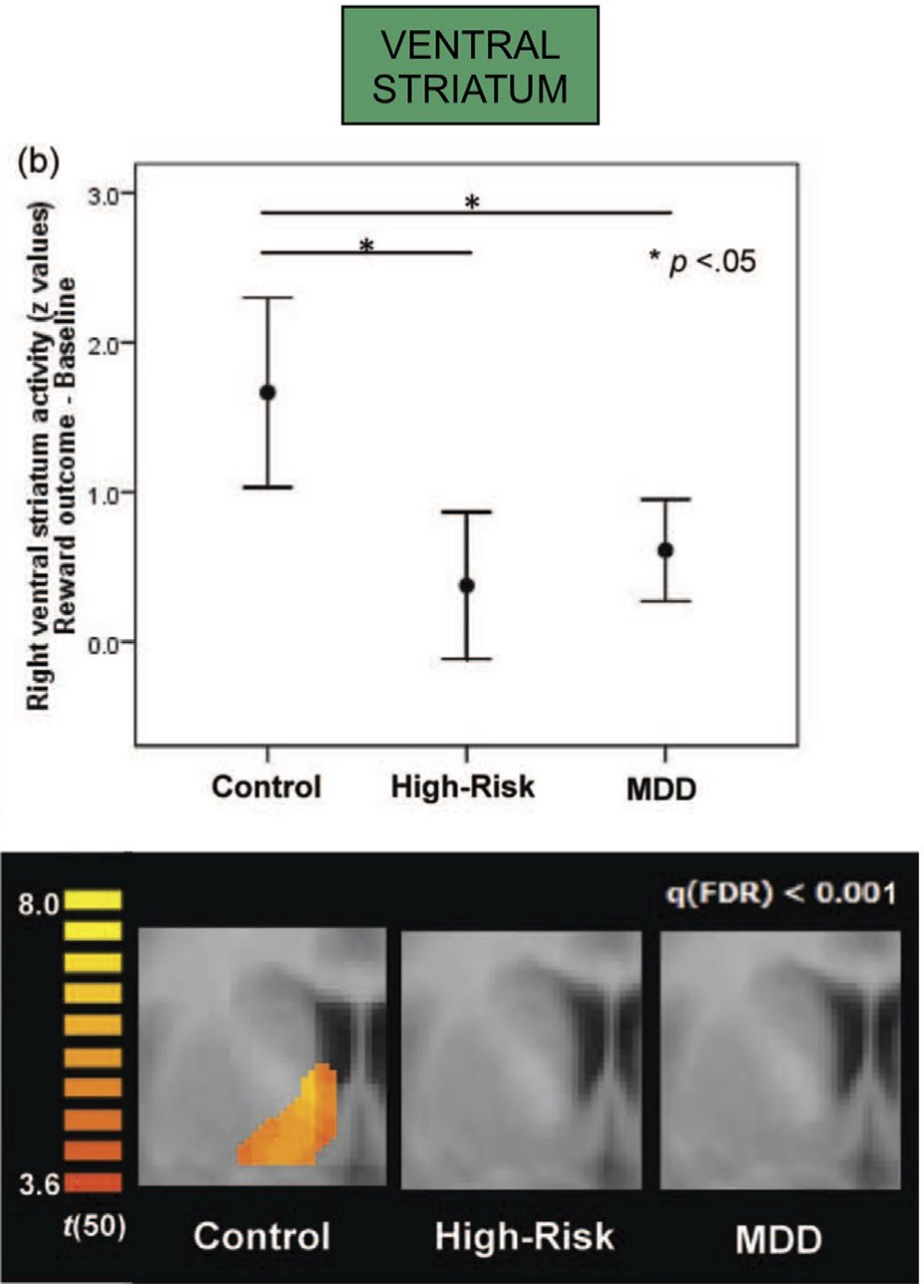
Both major depressive disorder (MDD) and high-risk groups (with current depression in mother but not in adolescent) show attenuated right ventral striatum activation in response to a standard reward outcome during functional MRI scanning (both *p < .05; error bars depict 95% confidence interval); activation for the reward outcome versus baseline contrast within the ventral striatum region of interest, presented at false-discovery-rate-corrected q < .001; the activations are shown at y = 5. Adapted from (117). Used with permission.

Depression is likewise associated with differences in function of dopamine-related regions. Both depressed adolescents, as well as non-depressed adolescents whose mothers were currently depressed, both showed diminished activation of the ventral striatum in a reward-based functional MRI task (117) (**Figure 4**). Furthermore, activation of the ventral striatum in the adolescents was inversely correlated with the mother’s—and not the adolescent’s—depression scores, suggesting that maternal depression may be contributing to an abnormal reward response in the offspring. Taken together, depression, novelty-seeking, and risk-taking behavior have been associated with increased susceptibility to addiction (**Figure 1**).

### Glucocorticoid-Related Stress-Response System Pathways

Glucocorticoids (cortisol in primates) are steroid hormones that contribute to the physiological stress response. A cascading set of neurotransmitters and hormones involved in this organization is collectively described as the hypothalamus–pituitary–adrenal (HPA) axis. Psychological or physical stress triggers the release of corticotrophin-releasing factor (CRF) in the hypothalamus, which binds to receptors in the pituitary to promote ACTH release. ACTH is then transported to the adrenal glands, resulting in secretion of the glucocorticoid stress hormone. Once released, glucocorticoids activate glucocorticoid receptors, which suppress further synthesis and release of CRF and ACTH, thereby providing negative feedback inhibition of the HPA system and restoring homeostasis.

In humans, decreased responsiveness, sensitivity, and synchrony in early caregiving have been correlated with prolonged or exaggerated increases in cortisol in response to stress (118, 119), whereas secure parental attachment has been associated with lower cortisol levels in response to stress (120) (**Figure 1**). However, in cases of more severe early deprivation or maltreatment, patterns of HPA responsiveness have been mixed, perhaps due to the complex nature of maltreatment and the co-occurrence with psychiatric disorders. Affected individuals may undergo a transition from early hypercortisolism to later hypocortisolism due to frequent and persistent adverse experiences (121, 122). This change may reflect adaptive down-regulation of the HPA system following chronic stress exposure, leading to flattened diurnal rhythms of cortisol secretion (with lower than normal daytime cortisol levels) (118, 123).

Rodents who experience diminished maternal care show increased DNA methylation of the glucocorticoid promoter region, which is associated with decreased glucocorticoid expression in the hippocampus and limited inhibitory feedback to stress (124). This results in elevated anxiety and fearfulness in adulthood and decreased exploratory behavior. These behavioral outcomes resemble signs and symptoms of anxiety, post-traumatic stress and addictive disorders in humans (125–127).

Early adverse experience is a potent pathway for the development of anxiety and trauma-related disorders later in life (90). Altered glucocorticoid and HPA responsiveness may contribute to the etiology of these disorders and mediate early adversity with later psychopathology and addiction (128, 129). Numerous studies connect stress dysregulation and HPA dysfunction to substance addiction (130, 131). Stress exposure may precipitate the onset of substance use, diminish the motivation to abstain, and heighten the risk for relapse, particularly in those with exaggerated HPA reactivity (131). This process may reflect the effects of a chronically activated HPA axis on enhanced striatal extracellular dopamine release, which may expose the reward system to the reinforcing properties of addictive substances (35, 132, 133). Disorders that are associated with HPA dysfunction, such as anxiety and trauma-related disorders, may serve as precursors to the development of substance addiction (128, 129, 134). These disorders may also modulate the progression of substance addiction, such that the illness course is typically more severe and persistent (135).

**Figure 5:**
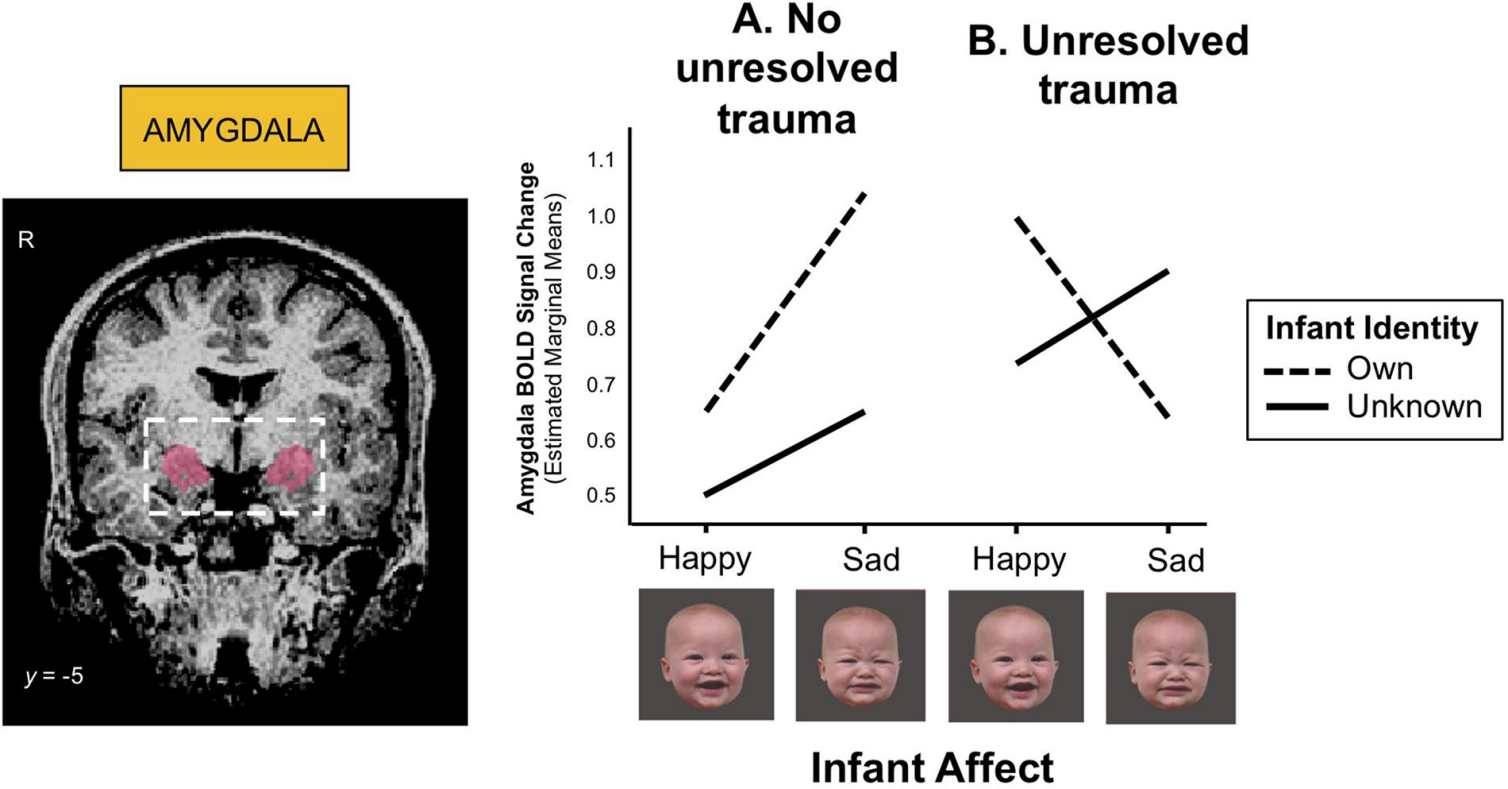
**(A)** Mothers with no unresolved trauma show a greater amygdala response to sad than happy infant faces (*z* = 3.00, *p* = 0.003), whereas **(B)** mothers with unresolved trauma show a blunted amygdala response to their own infant’s distress cues during functional MRI scanning (*z* = -2.38, *p* = 0.017). BOLD (blood-oxygen-level-dependent) signals were extracted from a bilateral amygdala mask (shown left) and submitted to mixed-effects linear regression analysis. Adapted from (138). Used with permission from Taylor & Francis Ltd.

The amygdala, which contributes importantly to the processing and regulation of emotions, connects with the striatum and prefrontal cortex (**Figure 2**), and its development has been associated with early life stress and trauma. Enlarged amygdala volumes have been seen in children exposed to chronic maternal depression (136), and in those raised in orphanages (137). In a study of mothers with unresolved trauma, based on the AAI, amygdala activation appeared to be “turned off” when these mothers viewed their own infant’s distressed face (138), despite responding similarly to mothers without unresolved trauma when viewing unknown infant faces (**Figure 5**). This suggests that unresolved childhood trauma may alter amygdala reactivity to salient attachment-based cues.

Substances of abuse, particularly depressants such as alcohol, benzodiazepines and cannabis, may be used to diminish the physiological and psychological effects of chronic stress as a result of childhood abuse (**Figure 1**). For example, one effect of alcohol is to dampen the neuroendocrine stress system by reducing peripheral glucocorticoid levels (140, 141). The effectiveness by which this is accomplished may itself lead to an increased susceptibility to addiction.

### Interactive Pathways

Although the prior discussion has focused on individual neuroendocrine systems, each system is interconnected with the other, rather than acting in isolation (5). For example, oxytocinergic neurons connect the hypothalamus with key dopaminergic brain nuclei, including the ventral tegmental area and the ventral striatum (142), as well as the amygdala. These systems all appear to play an important role in maternal behavior, pair-bond formation and social attachment (143, 144).

In humans, intranasal oxytocin enhances brain reward activation in both the ventral tegmental area and the ventral striatum (145). The effect of individual differences in oxytocin functioning on dopamine and other neuroendocrine systems, as well as the stress axis, may underlie differences in susceptibility to addiction (68). Likewise, exposure to drugs such as amphetamines may impair bonding and attachment *via* changes in oxytocin and dopamine neurotransmission in areas such as the ventral striatum and medial prefrontal cortex (146).

Oxytocin also appears to have a stress inhibitory effect, attenuating symptoms of anxiety and activation of the hypothalamic–pituitary–adrenal axis, especially with regard to substance abuse and withdrawal (147–149). One model proposes that oxytocin may attenuate stress and addiction by shifting the preference for novelty and reward seeking toward a greater appreciation for familiarity and attachment (69). As noted previously, early life stress, such as *via* maternal separation, may also effect dopamine functioning, from neuronal development, dopamine signaling and receptor expression, to addiction behaviors (3).

**Figure 6:**
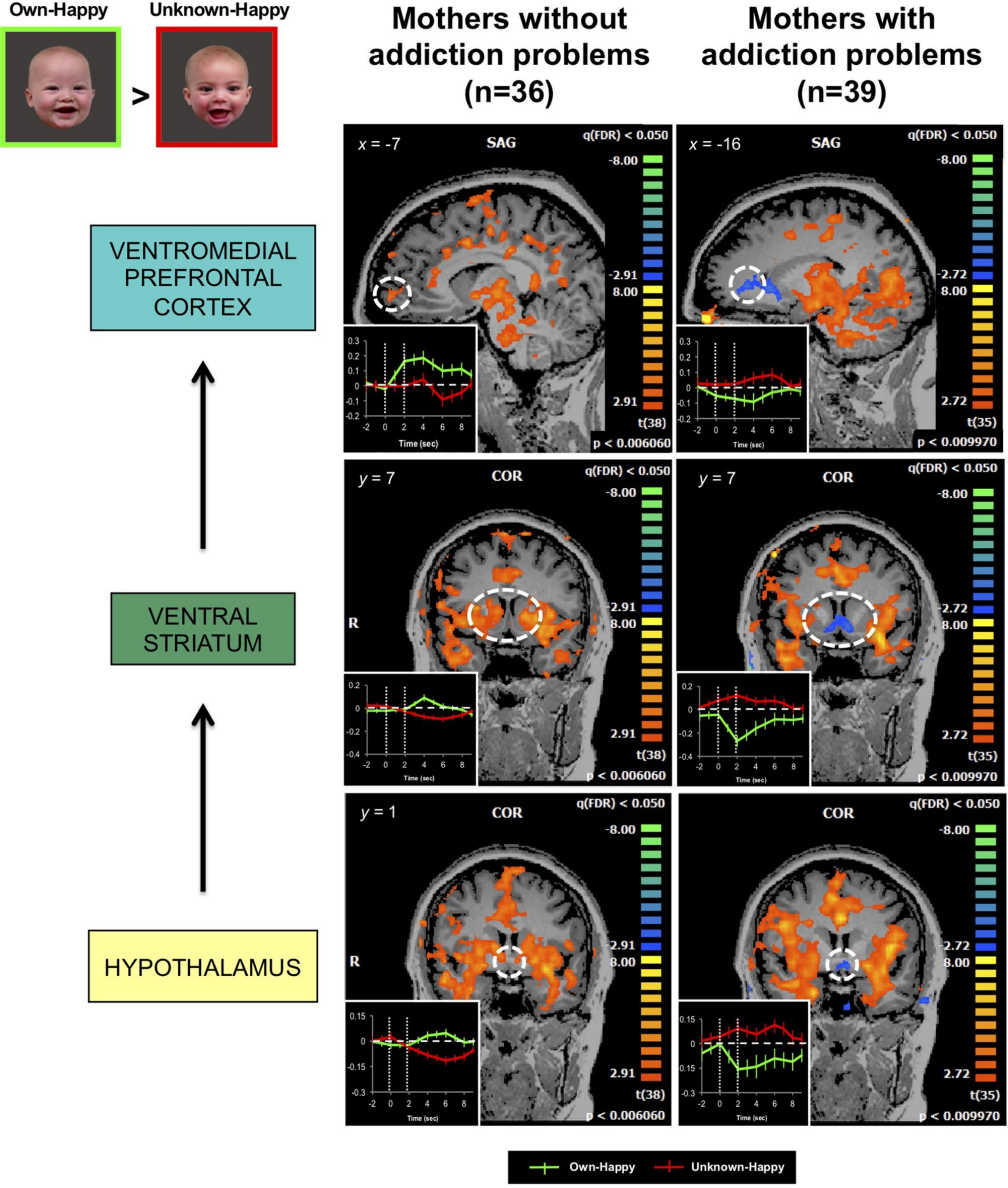
In response to own- vs. unknown-infant happy faces (OH > UH), mothers with addiction problems show deactivation in the hypothalamus, ventral striatum, and ventromedial prefrontal cortex, regions wherein strong activation has been observed in mothers without a history of substance use (random effects analysis; FDR-corrected *p* < 0.05). Inset shows brain response time courses extracted from the peak voxels in each specified region (hashed line circle), after presentation of own-happy (green plot) and unknown-happy (red plot) infant face cues between 0 and 2 seconds. Adapted from (139). © 2017 Wiley Periodicals, Inc. Used with permission.

Our own published work has demonstrated that mothers with drug addiction problems show a different brain response pattern in both dopamine reward and oxytocin-associated affiliation pathways, including the hypothalamus, ventral striatum and ventromedial prefrontal cortex, when viewing pictures of their own infant’s smiling face (139) (**Figure 6**). Instead of showing an increased response in these brain regions, as demonstrated in non-substance using mothers (150), the response in mothers with addiction problems was diminished, compared with the responses to unknown infant faces.

Thus, all three of these neuroendocrine systems may have interactive effects on attachment and the subsequent susceptibility to addiction.

## Conclusion

In this review paper, we have focused on three interconnected neuroendocrine pathways which, to some extent, may be programmed by early life experience and related to patterns of childhood attachment. Multiple overlapping adverse childhood experiences, ranging from traumatic abuse to absence of nurturant care and neglect, may have a profound impact on the development of secure attachment and on each of these three biological systems: the dopamine-related reward system, the oxytocin-related affiliation system, and the glucocorticoid-related stress response system. Other factors may also contribute to risk, including genetic differences, other neuroendocrine systems such as serotoninergic and glutamatergic pathways, and the effect that substance abuse itself may have on brain functioning and ongoing development.

We are currently working to determine whether differences in brain responses in mothers with addiction problems are related to drug use *per se*, as proposed in the brain disease model of addiction (10), or more fundamental underlying conditions, such as unresolved childhood trauma, insecure attachment, or other psychological or socio-demographic factors. A focus on attachment and developmental pathways may be important in delivering optimal treatment for drug-exposed mothers, as seen in some notable evidence-based recovery programs (151–153), as well as identifying key targets for early intervention and prevention efforts. By employing a lifespan developmental perspective, we may most appropriately address and target the intergenerational risk of substance use and addiction, and provide more hope for future generations.

## Author Contributions

All authors contributed to the formulation of the review paper topic. LS, CM, and SK drafted the original manuscript, and the other authors provided additional contributions and critical feedback.

## Conflict of Interest

MP has received financial support or compensation for the following: he has consulted for RiverMend Health, Game Day Data, the Addiction Policy Forum, and Opiant Pharmaceuticals; has received research support from Mohegan Sun Casino and the National Center for Responsible Gaming; and has consulted for gambling and legal entities on issues related to addictive disorders.

The remaining authors declare that the research was conducted in the absence of any commercial or financial relationships that could be construed as a potential conflict of interest.
